# Hospital Attendants and Male Servants

**Published:** 1905-01-21

**Authors:** 


### HOSPITAL ATTENDANTS AND MALE SERVANTS.

"ANOTHER HOSPITAL SECRETARY" writes: I have just seen the letter from "A Hospital Secretary" in your last week's number, and thought I would like to relate my experience.

For some years a license tax was paid by the hospital with which I am connected for its porter, but knowing that hospitals do not usually pay such licenses I applied to the Surveyor of Taxes for the district for exemption. He told me he could not deal with the matter and advised me to appeal direct to Somerset House. This I did, giving full particulars of the porter's duties and pointing out that he was not kept as a luxury but as a necessity. I am pleased to state that the Inland Revenue authorities wrote back very courteously, and not only stated that the claim should be withdrawn, but that no further demand for the payment of the license should be made in future.

This happened 10 years ago and I have had no trouble since. I cannot help feeling, therefore, that if your correspondent acts in a similar manner his hospital will be relieved of the payment of such an unjust tax.

